# Mycobacteriosis in slaughter pigs from South Africa from 1991 to 2002: *Mycobacterium* spp. diversity and *Mycobacterium avium* complex genotypes

**DOI:** 10.3389/fmicb.2023.1284906

**Published:** 2023-11-16

**Authors:** Nomakorinte Gcebe, Rian Ewald Pierneef, Anita Luise Michel, Motlatso Tiny Hlokwe

**Affiliations:** ^1^Bacteriology Laboratory, Agricultural Research Council–Onderstepoort Veterinary Research, Pretoria, South Africa; ^2^Agricultural Research Council–Biotechnology Platform, Pretoria, South Africa; ^3^Department of Biochemistry, Genetics and Microbiology, University of Pretoria, Pretoria, South Africa; ^4^Centre for Bioinformatics and Computational Biology, University of Pretoria, Pretoria, South Africa; ^5^Microbiome@UP, Department of Biochemistry, Genetics and Microbiology, University of Pretoria, Pretoria, South Africa; ^6^Department of Veterinary Tropical Diseases, Faculty of Veterinary Science, University of Pretoria, Pretoria, South Africa

**Keywords:** mycobacteriosis, *Mycobacterium avium* complex, MLVA, non-tuberculous mycobacteria, porcine lymphadenitis, zoonosis

## Abstract

**Introduction:**

*Mycobacterium avium* complex (MAC) bacteria are the most prominent etiological agents of lymphadenitis in pigs. *M. avium* subspecies *hominissuis* (MAH) is a member of MAC and has been reported in many parts of the world to be the most prevalent non-tuberculous mycobacteria (NTM) to cause mycobacteriosis in humans, mainly in children. Thus, the economic and zoonotic impact of MAC species are increasingly being recognized. In South Africa, little is known about the distribution of NTM and the molecular epidemiology of *M. avium* in pigs.

**Materials and methods:**

In this study, lymph nodes including mandibular, mesenteric, submandibular, and retropharyngeal, with tuberculosis-like lesions were collected during routine meat inspection of slaughter pigs with no disease symptoms (*n* = 132), between 1991 and 2002. These pigs were slaughtered at 44 abattoirs distributed across seven of the nine South African provinces. Mycobacterial culture, polymerase chain reaction (PCR), and sequencing of the *Mycobacterium* specific 577 bp 16S rRNA gene fragment were performed for species and subspecies identification.

**Results:**

The majority of the isolates (each per sample); 114 (86.4%) were identified as MAH, 8 (6%) as MAA*/M. avium* subsp. *silvaticum*, 4 (3%) were *Mycobacterium tuberculosis*, 2 (1.5%) as *Mycobacterium intracellulare*, and 1 (0.75%) as *Mycobacterium bovis.* The other isolates were identified as *Mycobacterium lentiflavum* (0.75%), *Mycobacterium novocastrense* (0.75%), and a *Micrococcus* spp. (0.75%). Using an eight-marker MLVA typing tool, we deciphered at least nine MIRU VNTR INMV types of MAH and MAA.

**Discussion:**

Identification of known zoonotic mycobacteria, including MAH, MAA, *M. intracellulare, M. bovis*, and *M. tuberculosis*, from slaughter pigs has a potential public health impact and also strengthens recognition of the potential economic impact of MAC. This study has also for the first time in South Africa, revealed MAC MIRU VNTR INMV genotypes which will aid in the future epidemiological investigation of MAC in South Africa.

## Introduction

The economic and potential zoonotic impact of porcine mycobacteriosis is increasingly being recognized world-wide (Johansen et al., 2014). Members of the *Mycobacterium avium* complex (MAC), including *M. avium* subsp. *avium* (MAA), *M. avium* subsp. *silvaticum* (MAS), *M. avium* subsp. *hominissuis* (MAH), and *Mycobacterium intracellulare* are the most prominent etiological agents of porcine mycobacteriosis with MAH being the most prevalent opportunistic pathogen of humans and porcine (Domingos et al., 2009; Johansen et al., 2014).

Porcine mycobacteriosis may be chronic and characterized by inflammatory reactions in various body parts but mostly in the digestive system. Calcification of tubercles, inflamed lymph nodes (lymphadenitis) and tuberculosis-like granulomas are the most common features of mycobacteriosis in pigs (Agdestein et al., 2014). Mycobacterial infection in pigs is mainly asymptomatic; as such, lesions are mostly detected during slaughter (Polaček and Aleksić-Kovačević, 2016). Some studies have reported clinical signs-like abortion and wasting due to MAH in pigs (Wellenberg et al., 2010; Eisenberg et al., 2012). This causes a significant economic burden as the meat from animals with lymphadenitis is considered unfit for human consumption, and may be condemned. Likewise, abortion decreases productivity and lead to economic losses for farmers. On the other hand, some studies have reported infection of pigs with MAC and other mycobacteria, to present no detectable clinical signs and the granuloma appear very minute (Agdestein et al., 2011, 2012). As such, pigs infected with mycobacteria can be missed during inspection at slaughter and the meat may enter the human food chain posing a serious public health threat especially to the immuno-compromised individuals, children, and the elderly (Polaček and Aleksić-Kovačević, 2016). Despite these findings, meat inspection is still an important practice for identification of pig mycobacterial infections in order to mitigate the risk of human exposure. In humans, MAH may cause pulmonary or soft tissue infections and cervical lymphadenitis in children (Ichikawa et al., 2009). A study in Japan reported pulmonary infection of HIV negative and positive patients with MAH (Ichikawa et al., 2009).

Non-tuberculous mycobacteria (NTM) other than MAC, including *Mycobacterium palustre, Mycobacterium bohemicum, Mycobacterium heckeshornense, Mycobacterium malmoense, Mycobacterium haemophilum, Mycobacterium szulgai, Mycobacterium kansasii, Mycobacterium scrofulaceum, Mycobacterium simiae, Mycobacterium gordonae, Mycobacterium terrae*, and *Mycobacterium xenopi* have also been detected from infected pigs (Cvetnić et al., 2007; van Ingen et al., 2010; Muwonge et al., 2012a). These NTM are also known to be opportunistic pathogens in humans (Tortoli et al., 2003).

The most prevalent pathogens associated with tuberculosis-like lesions in pigs are MAC, followed by *Mycobacterium tuberculosis, Mycobacterium bovis*, and *Rhodococcus equi* (Muwonge et al., 2012b; Cardoso-Toset et al., 2015). However, some other pathogens-like *Corynebacterium, Streptococci, Staphylococci, Enterococci*, and *Pasteurella* have been isolated from tuberculosis-like lesions in pigs (Cardoso-Toset et al., 2015; Scherrer et al., 2020).

In general, mycobacteria are currently not considered as significant food borne pathogens. This is despite reports from different countries about isolation from infected slaughter pigs, of members of MAC, NTM other than MAC and members of *M. tuberculosis* complex (MTBC) as well as isolation of MAC from muscle of infected pigs and in lymph-nodes without any visible lesions (Slana et al., 2010; Klanicova et al., 2011; Johansen et al., 2014). With little or no evidence of animal to human transmission of NTM including MAC, the environment is still considered the source of infection for both pigs and humans. However, research has now focused on molecular characterization of human and porcine MAC isolates with the aim to detect interspecies transmission patterns and the potential zoonotic nature of MAC. Outcomes of some of these studies indicated a close relationship between porcine and human isolates and therefore the zoonotic potential of MAC should not be ignored (Radomski et al., 2010; Tirkkonen et al., 2010; Ichikawa et al., 2015). Molecular genotyping methods for epidemiological investigation of MAC species include the restriction fragment length polymorphism (RFLP) with IS*1245*, IS*1311*, and IS*900* as markers (Johansen et al., 2007; Domingos et al., 2009). In recent years, multilocus variable number of tandem repeat analysis (MLVA) has been developed and is increasingly used for typing of MAC isolates (Thibault et al., 2007; Radomski et al., 2010). This method is based on the identification of mycobacterial repetitive elements, referred to as a mycobacterial interspersed repetitive unit, which results in a variable number of tandem repeats (MIRU-VNTR). A panel of eight MIRU-VNTR markers has been identified as a suitable tool to discriminate isolates and to study the genetic variability of the strains within the MAC. However, 7, 16, and 20 loci genotyping assays are also available (Inagaki et al., 2009; Leao et al., 2014).

In South Africa, earlier studies have reported isolation of *M. intracellulare* from slaughter pigs (Kleeberg, 1981; Nel, 1981). In addition, *M. bovis* was also isolated from the tissues of a slaughter pig (Hlokwe et al., 2014). This was the second report in the past decade that *M. bovis* has been isolated from pigs in South Africa, mainly due to the fact that in South African commercial farming, pigs and cattle are rarely kept together (Hlokwe et al., 2014). Other than these, there are no reports on the detection of mycobacteria from slaughter pigs in the country. The commercial pig industry in South Africa is still relatively small at 260,000 tons of pork produced in 2018 but signals a annual increase of 2.5%. There are approximately 400 pork producers and 19 stud breeders (DAFF, 2019). South Africa also has thousands of subsistence and small-scale farmers producing pork for families and communal use. South Africa despite being a net importer, exports pork to Namibia, Mauritius, and Mozambique (South Africa.co.za; 2018^1^). Therefore, investigation of zoonotic diseases-like mycobacterioses is of importance for public health and economic stability.

In this study we report mycobacterial infection of pigs with MAC, NTM other than MAC, *M. bovis* and *M. tuberculosis*, detected during slaughter between 1991 and 2002 in South Africa. We also report for the first time, the different strain types of MAC bacteria as revealed by MLVA typing.

## Materials and methods

### Ethics statement

This work was carried out from samples submitted for routine diagnostic purposes in the ARC-OVR’s Tuberculosis Laboratory: a Veterinary Laboratory of South Africa approved by the Department of Agriculture, Land Reform and Rural Development (DALRRD) in compliance with the requirements of the Animal Diseases act no. 35 of 1984 as well as DAFF/DALRRD 001, 002, and 012 procedures. The Animal Ethics Committees of the ARC-OVR (AEC 22.07) approved the study.

### Sample origin

**FIGURE 1:**
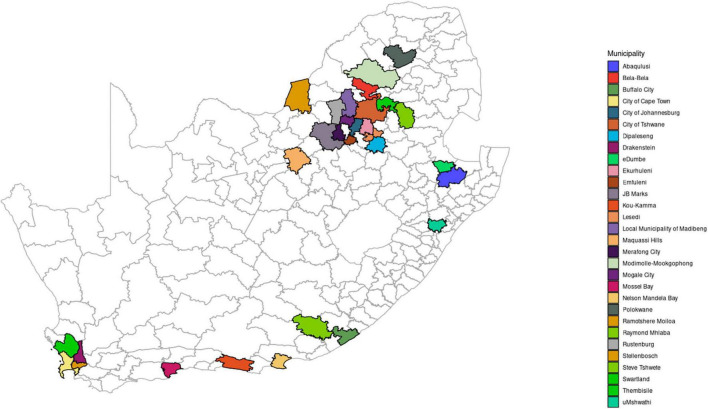
Map of South Africa showing administrative regions and illustrating the seven sampling sites (provinces): Mpumalanga, Gauteng, Limpopo, North West, Western Cape, Eastern Cape, and KwaZulu-Natal.

Lymph node samples (mesenteric, mandibular, submandibular, and retropharyngeal) were collected from slaughter pigs (*n* = 132) during routine meat inspection based on the observation of granulomatous lesions. The slaughter pigs were sampled at different slaughter houses (*n* = 44) in seven of the nine provinces of South Africa during regular meat inspection by Veterinary Public health officials between 1991 and 2002. These provinces include Western Cape, Eastern Cape, KwaZulu-Natal, Gauteng, North West, Limpopo, and Mpumalanga (Figure 1). The samples were submitted to the TB laboratory for diagnosis of suspected Mycobacterium infection including tuberculosis.

### Macroscopic examination of lymph nodes and culture

Lymph nodes to be processed for mycobacterial isolation were incised and examined macroscopically. The presence of granuloma, and lesions typical of calcification and caseous necrosis was recorded (Table 1).

**TABLE 1:** Diversity of *Mycobacterium* species isolated from different lymph nodes of slaughter pigs.

*Mycobacterium* species/subspecies	Mycobacterium count (%)	Lymph node
*M. avium* subsp. *avium*	8 (6)	Mesenteric, mandibular, submandibular
*M. avium* subsp. *hominissuis*	114 (86.4)	Mesenteric, mandibular, submandibular retropharyngeal
*M. bovis*	1 (0.75)	Mesenteric
*M. intracellulare*	2 (1.5)	Mesenteric
*M. lentiflavum*	1 (0.75)	Mesenteric
*M. novocastrense*	1 (0.75)	Mandibular
*M. tuberculosis*	4 (3)	Mesenteric, mandibular
*Micrococcus* spp.*	1 (0.75)	Mesenteric
Total	132	

*Non-*Mycobacterium* species.

Samples were processed for mycobacterial isolation as per OIE/WHOA guidelines (OIE, 2009). Briefly, approximately 5 g of tissue samples were first decontaminated on their surfaces for 1 min in 2% HCl and were cut into small pieces and then homogenized at 4,500 rpm in 100 ml of sterile distilled water using the Ultra-Turrax^®^ homogenizer (Separation Scientific SA). Two aliquots of 7 ml of the homogenate were each decontaminated with an equal volume of 2% HCl and 7 ml of 4% NaOH respectively for 10 min at room temperature. Following centrifugation, 7 ml of sterile distilled water was added to the pellet. The pellet was inoculated onto four Löwenstein-Jensen (LJ) media slopes supplemented with pyruvate (National Health Laboratories, South Africa), as well as two slopes supplemented with glycerol and an antibiotic cocktail of polymyxin B, ampho-tericin B, carbenicillin, and trimethoprim (PACT) (Becton Dickinson, South Africa). Three of the slopes were incubated at 37°C and the other three at 45°C and monitored weekly for mycobacterial growth. When growth of bacteria was observed, based on morphology of mycobacterial colonies, individual colonies were selected for Ziehl–Neelsen staining. The acid-fast cultures were stored at −20 or −80°C for further analysis.

### DNA extraction

DNA was extracted as described previously, using the heating method (Gcebe and Hlokwe, 2017). Briefly individual colonies were suspended in 100 μl of sterile distilled water in a 2 ml micro-centrifuge and heated at 95–100°C for 10–25 min, on a heating block. The suspension was allowed to cool at 25°C for approximately 5 min followed by centrifugation at 13,000 rpm for 5 min. The supernatant was transferred to a clean micro-centrifuge tube and used a DNA template in subsequent downstream polymerase chain reaction (PCR) reactions.

### *Mycobacterium* species identification by PCR

Polymerase chain reaction amplification of the genus specific and *M. avium* specific 16S rRNA gene fragment using MYCGEN-F (5′-AGAGTTTGATCCTGGCTCAG-3′), MYCGEN-R (5′-TGCACACAGGCCACAAGGGA-3′) and MYCAV (5′-ACCAGAAGACATGCGTCTTG-3′) primers was performed for detection of MAC, as described by Wilton and Cousins (1992). Briefly, a 25 μl PCR reaction mixture containing 14 μl de-ionized water, 2 μl MgCl_2_ (25 mM), 1 μl dNTP mix (10 mM), 1 μl of 10× PCR buffer (160 mM) (Tris–HCl, MgCl_2_, Tween-20, (NH_4_)_2_SO_4_), 0.125 μl Taq DNA Polymerase (5 U/μl) (Supertherm™), 0.5 μl of each primer (50 pMol) and 5 μl of DNA template was prepared. Amplification were as follows: initial denaturation at 94°C for 5 min, followed by forty cycles of denaturation at 94°C for 30 s, annealing at 62°C for 3 min, and elongation at 75°C for 3 min.

For non-MAC isolates, PCR followed by sequencing of the 577 bp fragment of the mycobacterial 16S rRNA gene was performed for mycobacterial species identification (Harmsen et al., 2003; Gcebe et al., 2013). Sequencing of the forward strands was done at the DNA sequencing unit at Inqaba Biotechnologies, South Africa using an ABI sequencer (Applied Biosystem Inc.). Sequences were edited manually using BioEdit software, then analyzed using the NCBI BLAST^2^ for mycobacterial species identification.

Members of the MTBC, which could not be differentiated by 16S rRNA gene sequencing, were identified by PCR amplification of the regions of difference RD 4 and RD 9 as described by Warren et al. (2006).

### *Mycobacterium avium* complex subspecies identification

Subspeciation of all *M. avium* isolates was done on the basis of the presence or absence of *IS901* which is present in MAA and *M. avium* subsp. *silvaticum* and commonly absent in *MAH* in swine; and in *M. avium* subsp. *paratuberculosis.* In addition we amplified *IS1245* which is present as multiple copies in MAH and MAA and absent in *M. avium* subsp. *paratuberculosis* (Bartos et al., 2006). Primers: IS901-L 5′-GCGCTGAGTTCCTCGTAAT-5′ and IS901-R CTTCGATCTTGAGGCTGGA were used for amplification of the IS901 (Turenne et al., 2006), and IS1245 P40 GAATCCGCAGTTCCAGGTC and 1245 P41 GGTGAGCGATCACTCAAG for amplification of the IS1245 sequence (Johansen et al., 2005).

### *Mycobacterium avium* complex MIRU VNTR genotyping

Eight Variable Number of Tandem Repeat (VNTR) loci described by Thibault et al. (2007) were used to genotype all MAH and MAA isolates identified in this study. PCR amplifications with all primer sets were performed individually. These were 25 μl PCR mixtures each containing 1.5 μl of 25 mM MgCl_2_, 5 μl of 10× Buffer, 0.5 μl of dNTP mix (10 mM), 0.1 μl of Taq DNA Polymerase (5 U/μl) and 1.25 μl of each forward and reverse primers. PCR amplification was done by 40 cycles denaturation at 94°C for 30 s, annealing at 58°C and extension at 72°C for 30 s followed by final elongation step at 72°C for 7 min. PCR amplicons were separated on a 3% agarose gel and amplicon sizes were estimated by comparison with a 100 bp molecular weight marker (Inqaba Biotechnologies Pty, LTD). The VNTR values (copy numbers) were scored by visual approximation using the guidelines set, by Thibault et al. (2007). The resulting alleles of MIRU-VNTR loci were assigned according to a previously described allele-calling table and arranged to profiles known as INMVs in the MAC-INMV database.^3^ The genetic relationship among the MAC isolates were inferred from the MIRU-VNTR loci using R v.4.3.0^4^ implemented in RStudio v.2022.07.2.576.^5^ A distance matrix was calculated using the “daisy” function with the “gower” parameter specified to determine Gower distances with the R package “cluster” (Maechler, 2019). Minimum spanning trees were calculated using the “ape” package (Paradis and Schliep, 2019), with the “mst” function, and visualized using “igraph” (Csardi and Nepusz, 2006) and “ggnetwork” (Briatte et al., 2020).

## Results

### Diversity of *Mycobacterium* species and subspecies detected in slaughter pigs

Mycobacterium isolation was made from 131 of the 132 lymph nodes submitted, all with tuberculosis-like granulomatous lesions. The majority of the isolates; 114 (86. 3%) were identified as MAH, 8 (6%) as MAA*/M. avium* subsp. *silvaticum*, 4 (3%) were *M. tuberculosis*, 2 (1.5%) as *M. intracellulare*, and 1 (0.75) as *M. bovis.* The other isolates were identified each as *Mycobacterium lentiflavum* 1 (0.7%), *Mycobacterium novocastrense* 1 (0.75%), and *Micrococcus* spp. 1 (0.75%) (Table 1).

### Geographic distribution of *Mycobacterium* species

The majority of the isolates (47.7%) originated from slaughter pigs in 15 abattoirs from Gauteng province, followed by nine abattoirs in North West province (16.6%) and then the other provinces with KwaZulu-Natal recording the least of the isolates in this study (4.5%) (Table 2). From Gauteng province, abattoir Br and in North West province, abattoir In had the most pronounced isolates (22/63 and 10/22, respectively) compared to other abattoirs within the these provinces. There seem to be even distribution of MAC subspecies which were isolated from all the seven provinces and in addition, Gauteng, Western Cape, and Eastern provinces also recorded cases of *M. tuberculosis* as well as *M. intracellulare, Micrococcus* spp., and *Mycobacterium novocastrence*, respectively (Table 2). In addition to *M. intracellulare* that was also isolated from KwaZulu Natal, *M. bovis* and *M. lentiflavum* were also isolated from this province (Table 2).

**TABLE 2:** The distribution of *Mycobacterium* spp. isolated from slaughter pigs across the different provinces of South Africa.

Province and number of abattoirs (*n*)	Abattoirs (numbers of isolates per abattoir)	Total number of isolates/samples (%)	*Mycobacterium* species identification
Gauteng (15)	Abattoir Br (*n* = 22) Abattoir Ste (*n* = 12) Abattoir Cha (*n* = 07) 5× abattoirs (*n* = 3 each) 7× abattoirs (*n* = 1)	63 (47.7)	MAA (*n* = 5), MAH (*n* = 56), *M. intracellulare* (*n* = 1)*, M. tuberculosis* (*n* = 1)
North West (9)	Abattoir In (*n* = 10) 4× abattoirs (*n* = 2) 4× abattoirs (*n* = 1)	22 (16.6)	MAA (*n* = 2), MAH (*n* = 20)
Western Cape (6)	5× abattoirs (*n* = 2 each) 1× abattoir (*n* = 1)	11 (8.3)	MAH (*n* = 8), *M. tuberculosis* (*n* = 2), *Micrococcus* spp.* (*n* = 1)
Limpopo (3)	Abattoir Ma (*n* = 7) Abattoir Gr (*n* = 2) Abattoir Wa (*n* = 6)	14 (10.6)	MAH (*n* = 14)
Eastern Cape (4)	4× abattoirs (*n* = 2 each)	8 (6)	MAH (*n* = 6), *M. tuberculosis* (*n* = 1)*, M. novocastrense* (*n* = 1)
Mpumalanga (3)	2× abattoirs (*n* = 2) 1× abattoir (*n* = 3)	7 (5)	MAH (*n* = 7)
KwaZulu Natal (4)	Abattoir Ca (*n* = 3) 3× abattoirs (*n* = 1 each)	6 (4.5)	MAA (*n* = 1), MAH (*n* = 2), *M. bovis* (*n* = 1), *M. lentiflavum* (*n* = 1)*, M. intracellulare* (*n* = 1)
Total		132	

MAA, *Mycobacterium avium* subsp. *avium*; MAH, *Mycobacterium avium* subsp. *hominissuis*; *, non-*Mycobacterium*.

### Isolation trends of mycobacteria over the study period

The majority of cases (40%) were submitted in 1998, followed by submissions in 2000 (25%) while between 1993 and 1995, there were no cases submitted (Table 3). In 1992, the laboratory recorded the least number of cases (0.75%). MAC subspecies (MAH and MAA) were isolated throughout the submission period, while in 1991 different mycobacteria seem to have contributed to mycobacteriosis cases in slaughter pigs. These were in addition to MAC; *M. tuberculosis, M. bovis, M. lentiflavum, M. novocastrense* as well as *Micrococcus* species. *M. tuberculosis* was isolated during meat inspection in 1996.

**TABLE 3:** *Mycobacterium* spp. isolation trends over time (1991–2002).

Year of isolation	Number of isolates (%)	*Mycobacterium* spp.
1991	17 (12.9)	MAH, *M. tuberculosis, M. bovis, M. lentiflavum, M. novocastrense*, *Micrococcus* spp.
1992	1 (0.75)	MAH
1996	2 (1.5)	MAH and *M. tuberculosis*
1998	53 (40)	MAH, MAA, *M. intracellulare*
1999	9 (12.9)	MAH
2000	33 (25)	MAH, MAA
2001	8 (6)	MAA
2002	9 (12.9)	MAA, MAH
Grand total	132	

MAH, *Mycobacterium avium* subsp. *hominissuis*; MAA, *Mycobacterium avium* subsp. *avium*.

### *Mycobacterium avium* complex MIRU VNTR types: spatial and temporal distribution

*Mycobacterium avium* complex MIRU VNTR profiling revealed occurrence of nine known INMV types namely; INMV77, INMV74, INMV248, INMV13, INMV55, INMV43, INMV6, INMV184, and INMV9 in 122 MAH and MAA isolates. In addition, three potential novel INMV types were detected. Table 4 illustrates the spatial distribution of the INMV types. The most prevalent INMV type was INMV74 (45%) followed by INMV77 (26.2%), INMV248 (10.7%), INMV13 (8.2%), and INMV55 (4.1%), while INMV43, INMV6, INMV184, and INMV9 were the least isolated in the study each accounting for 0.8% of the total cases. MAH was represented in all the INMV types while MAA constituted of INMV74, INMV77, INMV13, and INMV248. Among the INMV types, INMV248 and INMV74 circulated in all the seven provinces. Other INMV genotypes were only in four (INMV77 and INMV55) and three (INMV13) provinces while the rest of the genotypes were seen in one province each (INV43, INMV9, and INMV6). Likewise, the three potential new genotypes were each isolated from North West province (*n* = 2) as well as Gauteng province (*n* = 1) (Table 4). In terms of temporal distribution, our results show that the most prevalent INMV types, i.e., INMV74 strain was equally distributed between the years 1998 and 2000 with sporadic cases detected in the years 1991, 1996,1999, 2001, and 2002; and INMV77 strain was found mostly in 1998, with sporadic cases seen in the years 1999–2002. All the INMV types except INMV6, INMV9, and INMV184 were circulating in 1998, including the potential novel strains (Table 4).

**TABLE 4:** Spatial and temporal distribution of the INMV strains of MAC.

INMV type	Years of isolation	Number of isolates (%)	MIRU-VNTR string	MAC subspecies	Geographic distribution
INMV77	1998, 1999, 2000, 2001, 2002	32 (26.2)	3 2 2 3 2 2 2 8	MAH, MAA	KZN, NW, GP, MP
INMV74	1991, 1996, 1998, 1999, 2000, 2001, 2002	55 (45)	3 3 3 3 2 2 2 8	MAH	LP, EC, GP, MP, WC, NW, KZN
INMV248	1991, 1998, 2000, 2001, 2002	13 (10.7)	1 2 3 3 2 2 2 8	MAH, MAA	EC, MP, NW, WC, GP, WC, LP
INMV13	1998, 1999, 2000, 2001, 2002	10 (8.2)	2 3 2 3 2 2 2 8	MAH, MAA	LP, NW, GP
INMV55	1991, 1992, 1998, 1999	5 (4.1)	2 2 2 3 1 2 2 8	MAH	WC, MP, EC, NW
INMV43	1998	1 (0.8)	2 2 2 2 1 2 2 8	MAH	GP
INMV9	1991	1 (0.8)	2 1 3 3 2 2 2 8	MAH	KZN
INMV6	2000	1(0.8)	3 2 3 3 2 1 2 8	MAH	LP
INMV184	1991	1 (0.8)	0 5 3 3 1 2 2 8	MAH	WC
New	1998	1 (0.8)	0 4 3 3 2 2 2 8	MAH	NW
New	1998	1 (0.8)	1 4 3 3 2 2 2 8	MAH	NW
New	1998	1 (0.8)	1 5 4 3 1 2 2 8	MAH	GP
Grand total		122			

MAA, *Mycobacterium avium* subspecies *avium*; MAH, *Mycobacterium avium* subspecies *hominissuis*; LP, Limpopo province; NW, North West province; GP, Gauteng province; WC, Western Cape province; MP, Mpumalanga province, KZN, KwaZulu-Natal province; EC, Eastern Cape province.

### Phylogenetic analysis

**FIGURE 2:**
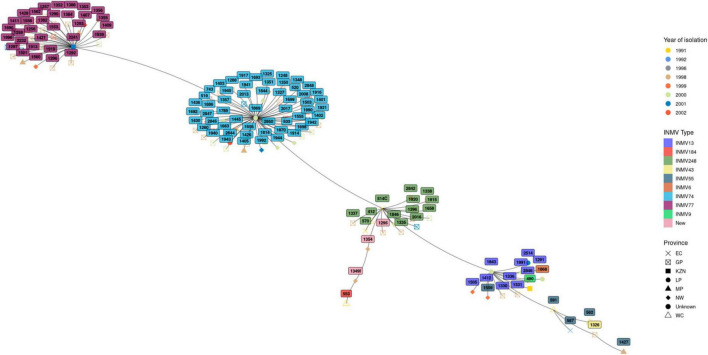
Minimum spanning tree showing clustering of INMV types of MAA and MAH, spatial and temporal distribution.

The genetic relationship among the isolates belonging to the different INMV types is illustrated in the minimum spanning tree in Figure 2. Overall four distinct clusters were observed with two major clusters representing isolates belonging to INMV74 and INMV77, while the other clusters contained isolates from the other INMV types. The two major clusters were distributed throughout the study years. One of the major clusters harboring strain INMV74 was distributed across all seven studied provinces while the second cluster occurred in four of the seven provinces.

## Discussion

In this study, the occurrence of Mycobacteria was assessed in slaughter pigs that showed tuberculous lesions during routine meat inspection in 44 slaughterhouses from seven provinces in South Africa, during an 11-year period (1991–2002). Morphological inspection of carcasses at slaughter houses constitute the first line investigation for food safety. Gross tuberculosis-like lesions in pigs are primarily situated in the lymph nodes of the head, respiratory and gastrointestinal tracts, mainly the mesenteric lymph nodes, less in mandibular tonsils, further in diaphragms and other organs (Agdestein et al., 2012*;* Kriz et al., 2014). In the current study, established pathogenic and opportunistic pathogenic mycobacteria of both humans and animals were isolated from mandibular, submandibular mesenteric and retropharyngeal lymph nodes showing gross tuberculosis like lesions, suggesting mycobacteriosis in the slaughter pigs. Isolation of MAC, including *M. avium* and *M. intracellulare* in 94% of the lymph nodes in this study, supports similar findings suggesting that MAC are the predominant etiological agents of porcine mycobacteriosis in several studies. A study in the Czechia reported MAC to be responsible for mycobacterial infection in 93% of infected pigs (Shitaye et al., 2006). Other studies in Europe reported similar findings, where in Switzerland, MAC was reported to be predominant (91%) of all mycobacteria isolated from the different lymph nodes of slaughtered pigs (Offermann et al., 1999). In the Netherlands, MAC were found to be responsible for infection in 54.2% of slaughter pigs (Komijn et al., 1999). Likewise, in Croatia, 96% of mycobacterial isolates from the lymph nodes of slaughtered pigs were reported to belong to MAC (Cvetnić et al., 2007). In Africa, studies conducted in Uganda also reported similar findings where (74%) of the *Mycobacterium* species isolated from lymph nodes of slaughter animals were MAC (Muwonge et al., 2012a). The current study is the first to report the detection of MAC from slaughter pigs in South Africa. Most studies of mycobacterial infection of animals in South Africa have focused mainly on cattle and buffalo as they are primary reservoirs of bovine tuberculosis, as well as other wildlife species (Hlokwe et al., 2014). Farming practices in South Africa are mainly commercial with approximately 400 farmers. The pig industry in South Africa has three main sectors including large-scale modern, efficient, intensive production units which have between 600 and 5,000 sow units; as well as medium and small scale commercial producers. In addition to commercial farming there are subsistence and small holder farmers producing pork for household and communal use (South Africa.co.za; 2018, see text footnote 1). The pigs slaughtered at abattoirs originated from commercial farms. Infection of the pigs with MAC could have been from the environment, i.e., shared water supply, soil as well as from contaminated peat and feed.

In the current study, within MAC, *M. avium* was predominant (92.4%) followed by *M. intracellulare* (1.5%). This finding further confirms reports from other studies of *M. avium* being the common etiological agent of porcine mycobacteriosis (Komijn et al., 1999; Stromerova and Faldyna, 2018).

Before the era of molecular methods which were not able to delineate MAA from MAH, MAA were thought to be responsible for most of porcine mycobacterial infections (Matlova et al., 2005). In this study, using IS*1234* and IS*901* as markers to differentiate between subspecies of *M. avium*, we showed that MAH was responsible for the majority of infections (86.4%) of the slaughtered pigs followed by MAA (6%). Some studies have however shown in some rare clinical cases in humans and bovine that MAH isolates harbored IS901 with ISMav6 mutations (Ichikawa et al., 2009; Scherrer et al., 2018). This finding further strengthens previous reports that among MA subspecies, MAH is the predominant mycobacteria to cause porcine mycobacteriosis (Barandiaran et al., 2015).

In addition to MAC, *M. bovis* was detected in a lymph node showing visible lesions. *M. bovis* is the main causative agent of tuberculosis in animals with a broad animal host range including both domestic animals and wildlife (Hlokwe et al., 2014). This is the third report of infection of pigs with *M. bovis* in South Africa (Hlokwe et al., 2014). The zoonotic nature of *M. bovis* makes exposure to this bacillus a public health risk in particular to immuno-compromised individuals (Muwonge et al., 2012b). Detection of *M. bovis* from pigs during slaughter has been reported from countries such as the Great Britain, Uganda, Argentina, and Nigeria (Barandiaran et al., 2011; Jenkins et al., 2011; Muwonge et al., 2012b; Bailey et al., 2013). Pig infection with *M. bovis* is rare in commercial farming systems due to the separation of pigs from cattle, which are the primary domestic reservoirs of bovine tuberculosis (bTB) (Hlokwe et al., 2014). In addition to MAC and *M. bovis*, we also detected *M. tuberculosis* from four slaughter pigs. Transmission of this pathogen to pigs may possibly be from consumption of contaminated uncooked garbage. Occurrence of this etiological agent of human tuberculosis poses a serious threat to human health, as abattoir workers might be exposed. Furthermore, if missed during inspection, the meat could enter the food chain. Studies from Egypt, Ethiopia, and Spain have reported the detection of *M. tuberculosis* from domestic pigs during slaughter (Mohamed et al., 2009; Arega et al., 2013; Cardoso-Toset et al., 2015).

*Mycobacterium lentiflavum* is an opportunistic human pathogen causing cervical lymphadenitis, therefore detection of this pathogen in slaughter pigs poses a potential public health risk (Tortoli et al., 2006). To the best of our knowledge, *M. lentiflavum* infection in pigs from South Africa is reported in this study for the first time. We also detected *M. novocastrense*, a rapidly growing opportunistic pathogenic NTM of humans which has been reported to cause pulmonary and wound infections in both healthy and immuno-compromised individuals (Shojaei et al., 2011). The current study is the first to report infection of pigs with this NTM.

Compared to other mycobacteria such as *M. bovis* and *M. tuberculosis*, the genetic diversity of MAC is not well documented. To understand the genetic diversity and molecular epidemiology of MAC in South Africa, we also set out experiments to genotype 122 isolates from this study using an eight loci MLVA typing assay. We, for the first time in the country, deciphered MAC INMV types and assigned the isolates to nine known genotypes/INMV types as well as three potential novel INMV types that are not available on the MAC-INMV SSR database (see text footnote 3). The most prevalent as well as the third most prevalent INMV types, i.e., INMV74 and INMV248 were widely distributed throughout the studied provinces, while other genotypes only occurred in certain provinces. Phylogenetic analysis of the MAH and MAA revealed four distinct clusters consisting of two major clusters made of INMV74 and INMV77, with the minor clusters consisting the remaining genotypes. These clusters seem to be distributed throughout the years of this study. In addition, the majority of isolates from these clusters were circulating in the years 1998 and 2000. Furthermore, all strains identified in this study, including the potential novel genotypes occurred in 1998. This could be attributed to the majority (40%) of cases submitted in that year, followed by (25%) submitted in the year 2000. Distribution of these two major clusters throughout the seven and four provinces respectively over an extended period of time could be due to movement of pigs across different provinces during exchange of breeding stock and spread by these animals in the receiving facility. Our retrospective data forms a baseline for future epidemiological studies of MAC, including human as well as animal cases in the South Africa.

## Conclusion

In conclusion, we have shown that MAH are the predominant etiological agents of porcine mycobacteriosis in South Africa confirming previous findings from other studies conducted in other countries. In addition, isolation of zoonotic pathogens, including *M. bovis* and *M. tuberculosis*, from slaughter pigs poses potential public health and economic risks. We also report infection of slaughter pigs with opportunistic human NTM spp. including *M. lentiflavum* and *M. novocastrense* which may also pose public health and economic risks. In addition we, deciphered MIRU VNTR types of MAC, which forms the basis for future epidemiological studies of this *Mycobacterium* in South Africa.

To date MAC species have not been considered an important food safety as well as food nutrition and security threat in South Africa. It should be noted that since 2002 there has not been reported cases of mycobacteriosis from slaughter pigs in South Africa. This may be due to improved bio-security measures within the breeding facilities or they are simply missed during routine inspection as the pigs may not show clinical sign and the granuloma may appear minute (Agdestein et al., 2011, 2012). Results from this study call for, in addition to routine inspection, resuscitation of active surveillance and monitoring programs of slaughter pigs for potential mycobacterial infections in the country.

## Data availability statement

The raw data supporting the conclusions of this article will be made available by the authors, without undue reservation.

## Ethics statement

The animal study was approved by the Agricultural Research Council-OVR Animal Ethics Committee. The study was conducted in accordance with the local legislation and institutional requirements.

## Author contributions

NG: Conceptualization, Formal analysis, Investigation, Writing – original draft, Writing – review and editing. REP: Formal analysis, Software, Writing – review and editing. ALM: Investigation, Writing – review and editing. MTH: Conceptualization, Formal analysis, Methodology, Writing – review and editing.

## Footnotes

1
https://southafrica.co.za/pig-industry-south-africa.html

2
https://blast.ncbi.nlm.nih.gov/Blast.cgi?

3
http://mac-inmv.tours.inra.fr/index.php?p=fa_ident

4
https://www.R-project.org/

5
https://rstudio.com
